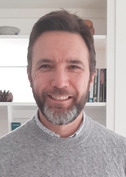# Copper‐Catalyzed Borylative Couplings with C−N Electrophiles

**DOI:** 10.1002/anie.202007251

**Published:** 2020-07-28

**Authors:** Fabien J. T. Talbot, Quentin Dherbassy, Srimanta Manna, Chunling Shi, Shibo Zhang, Gareth P. Howell, Gregory J. P. Perry, David J. Procter

**Affiliations:** ^1^ Department of Chemistry University of Manchester Oxford Road Manchester M13 9PL UK; ^2^ School of Material and Chemical Engineering Xuzhou University of Technology Xuzhou 221018 P. R. China; ^3^ Chemical Development Pharmaceutical Technology & Development, Operations AstraZeneca Macclesfield UK

**Keywords:** borocupration, copper, imine, multicomponent reaction, nitrile

## Abstract

Copper‐catalyzed borylative multicomponent reactions (MCRs) involving olefins and C−N electrophiles are a powerful tool to rapidly build up molecular complexity. The products from these reactions contain multiple functionalities, such as amino, cyano and boronate groups, that are ubiquitous in medicinal and process chemistry programs. Copper‐catalyzed MCRs are particularly attractive because they use a relatively abundant and non‐toxic catalyst to selectively deliver high‐value products from simple feedstocks such as olefins. In this Minireview, we explore this rapidly emerging field and survey the borylative union of allenes, dienes, styrenes and other olefins, with imines, nitriles and related C−N electrophiles.

## Graphical abstract

**Copper‐catalyzed** borylative multicomponent couplings, involving olefins and C−N electrophiles, constitute an efficient approach to high‐value amine and nitrile products. This Minireview assesses the progress in this rapidly emerging field and surveys the borylative union of allenes, dienes, styrenes, and related olefins, with imines, nitriles, and related electrophiles.

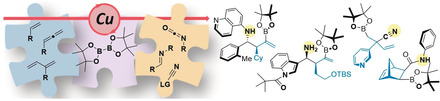

## 1 Introduction

Building molecules that contain nitrogen is of great importance: amines constitute 80 % of the bioactive targets used in drug discovery,^[1]^ and the number of nitrile‐containing drugs has been steadily increasing in recent decades.^[2]^ In addition, the versatile reactivity of nitrogen‐containing functional groups make them highly useful building blocks in synthesis. Likewise, boron‐containing compounds are involved in 11 % of C−C bond forming reactions in process chemistry.^[3]^ Thus, the union of nitrogen and boron‐containing functional groups in defined molecular scaffolds is highly sought after. Indeed, to venture into underexplored regions of chemical space, novel disconnections of targets containing these important functionalities are needed.^[4]^ Multicomponent reactions (MCRs) figure amongst the most promising strategies for addressing this challenge as they transform readily available feedstocks into complex structures in a single step.^[4b]^ In addition, due to their favorable atom and waste economies,^[5]^ MCRs are ideal for the synthesis of bioactive targets.^[6]^

**Scheme 1:**
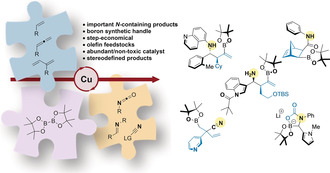
Copper‐catalyzed borylative multicomponent reactions involving olefins and C−N electrophiles. LG=leaving group. TBS=*t*‐butyldimethylsilyl.

The need to replace precious metals with more abundant and less toxic elements, such as copper, is a pervading theme in contemporary synthesis.^[7]^ Since the first reports by Hosomi and Miyaura at the turn of the century,^[8]^ the copper‐catalyzed borylation of C−C multiple bonds, along with the powerful extension of this methodology in MCRs, has been extensively studied.^[9]^ The aim of this Minireview is to highlight recent advances in copper‐catalyzed borylative MCRs involving C−N electrophiles and olefins for the synthesis of highly functionalized amines, nitriles and other nitrogen‐containing products (Scheme 1).

## 2 Mechanistic Aspects in Copper‐Catalyzed Borylative Couplings

**Scheme 2:**
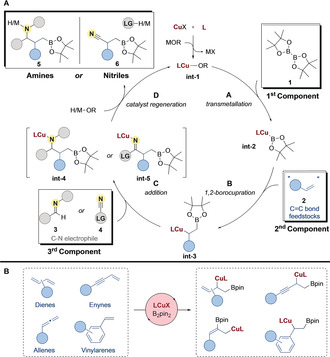
General mechanism for multicomponent Cu‐catalyzed borylative couplings.

Organocopper reagents can be generated in situ through borocupration of olefins, and then intercepted by C−N electrophiles (Scheme 2 A). A copper salt is first transformed into a copper(I) alkoxide complex **int‐1** by treatment with base.^[10]^ Transmetallation then occurs between **int‐1** and a diboron reagent **1**, typically via σ‐bond metathesis (step **A**).^[11]^ NHCs and phosphines are popular ligands in these processes, in fact, the first isolated copper‐boryl complexes **int‐2** featured NHCs as stabilizing ligands.^[12]^ 1,2‐Borocupration of an activated olefin **2** (internal, non‐conjugated olefins are unreactive)^[13]^ with **int‐2** produces the borylated organocopper complex **int‐3** (step **B** and Scheme 2 B).^[14]^ The regioselectivity and stereoselectivity of the borocupration is not easy to predict as both kinetic and thermodynamic factors must be taken into account.^[13]^ Furthermore, isomerization^[15]^ and rearrangement^[14]^ can lead to epimerization^[16]^ of the organocopper. Finally, reaction with a suitable electrophile **3/4** delivers the product **5/6** (step **C**/**D**). The catalyst is regenerated using an equivalent of base and diboron, or by protonation of the product with an alcohol (step **D**). This simplified mechanism encompasses many of the transformations in this Minireview. As steps **B** and **C** determine the regio‐ and stereoselectivity of these reactions, they will be highlighted in the text when mechanistic evidence is available.

## 3 Copper‐Catalyzed Borylative Couplings with Imines

Classical syntheses of amines often involve the addition of organometallic reagents to imines.^[17]^ However, these reactions suffer from the inherent limitations associated with pre‐formed organometallic reagents: cryogenic temperatures, air and moisture sensitivity, safety risks and pre‐functionalized starting materials. Copper species are well known to modulate the reactivity of organometallic reagents^[18]^ and can induce stereocontrol in additions to ketimines^[19]^ and aldimines.^[20]^ The use of copper catalysts to generate organocopper species in situ in MCRs is an attractive solution to the problems associated with stoichiometric organometallic reagents in amine synthesis.

### 3.1 Allenes

**Scheme 3:**
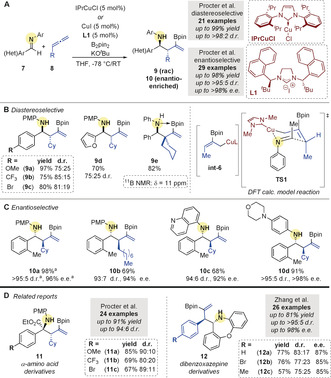
A) Procter's Cu‐catalyzed borylative coupling of aldimines and allenes. B) Diastereoselective variant. C) Enantioselective variant. [a] 2 gram scale reaction (1 mol % CuI/**L1**). D) Procter's and Zhang's coupling of allenes with imine derivatives.

In an effort to circumvent the need for pre‐formed allylmetal reagents in additions to imines,^[21]^ Procter and co‐workers^[22]^ reported the first multicomponent copper‐catalyzed borylative coupling of imines and allenes in 2016. Homoallylic amines **9** were formed from the addition of allylcopper complexes (e.g. **int‐6**), formed in situ by borocupration of 1‐monosubstituted or 1,1‐disubstituted allenes **8**, to aldimines **7** (Scheme 3 A). A range of substituents on the imine were well tolerated, including electron‐rich and electron‐deficient (hetero)aromatic groups (to give **9 a**–**e**, Scheme 3 B). Interestingly, X‐ray and ^11^B NMR analysis of the products revealed a donor‐acceptor interaction between the amine and the Bpin moiety (as illustrated in **9 e**). A density functional theory (DFT) study was performed to rationalize the observed *anti*‐diastereoselectivity. After considering various possibilities, a lowest energy pathway featuring a 6‐membered, Zimmerman–Traxler‐type transition‐state **TS1** from (*Z*)‐allylcopper species **int‐6** and aldimine **7** was proposed.

Procter and co‐workers^[23]^ also reported an enantioselective variant of the reaction. By using the enantiopure NHC precursor **L1** with CuI (5 mol %), excellent levels of stereoinduction and good to excellent yields were obtained (Scheme 3 C). Importantly, scalability was achieved using a low catalytic loading (1 mol %) on a 2 gram scale, affording **10 a** in almost quantitative yield, and with excellent diastereo‐ and enantioselectivity.

These reports triggered the development of similar MCRs involving imine derivatives. For example, the Procter group^[24a]^ used the approach to prepare quaternary α‐amino acid derivatives **11** from ketiminoesters (Scheme 3 D), for which an enantioselective variant has been reported by Chen et al.^[24b]^ In addition, Zhang and co‐workers^[25]^ realized the enantioselective coupling of arylallenes with cyclic imines to access functionalized dibenzo‐1,4‐oxapines **12** (Scheme 3 D).

**Scheme 4:**
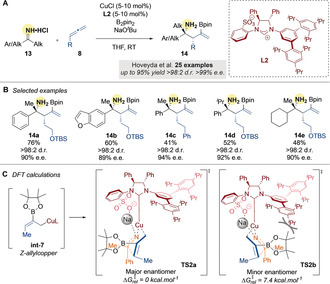
Hoveyda's Cu‐catalyzed borylative couplings of N−H ketimines and allenes.

In 2017, Hoveyda and co‐workers^[26]^ developed an enantioselective, copper‐catalyzed borylative coupling of allenes and N−H ketimines (Scheme 4 A). The instability of ketimine electrophiles was cleverly managed by using the HCl salt of N−H ketimines **13**, which were prepared through addition of an organolithium reagent to the corresponding nitrile and subsequent acidification. Using an enantiopure NHC ligand **L2** with CuCl (5–10 mol %), the ketimine salts were combined with 1‐substituted allenes **8** and B_2_pin_2_ to give the desired products in good to excellent yields and excellent diastereo‐ and enantioselectivities (Scheme 4 B). In agreement with the studies of Procter and co‐workers (Scheme 3 B), DFT calculations supported a mechanism involving a six‐membered transition state **TS2** (Scheme 4 C). They postulated that a combination of N→Na coordination and steric repulsion between the ligand and the Bpin moiety accounts for the high enantioselectivity of the transformation.

### 3.2 Vinylarenes

**Scheme 5:**
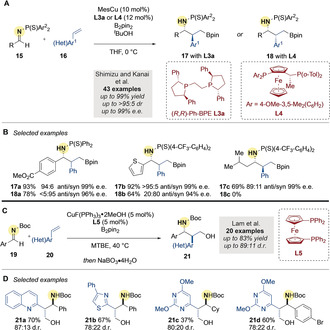
A,B) Kanai's enantioselective Cu‐catalyzed borylative couplings of aldimines and vinylarenes. C,D) Lam's Cu‐catalyzed borylative coupling of vinylazaarenes with imines. MTBE=methyl‐*tert*‐butyl ether.

In 2018, Kanai, Shimizu and co‐workers^[27]^ disclosed the first enantioselective copper‐catalyzed borylative coupling of aldimines **15** and vinylarenes **16** using mesitylcopper (MesCu) as a pre‐catalyst (Scheme 5 A).^[28]^ Notably, they were able to selectively access either *anti*‐ or *syn*‐diastereomeric products by varying the chiral ligand (**L3 a** or **L4**). A wide range of products was obtained in high yields and high enantiomeric ratios (Scheme 5 B). Provided that a large excess (10 equiv) of vinylarene was used, aliphatic imines were also suitable candidates in spite of their potential to tautomerize to enamines.

Prior to the report by the Kanai lab, Lam and co‐workers^[29]^ had disclosed a related borylative coupling of aryl‐substituted, *N*‐Boc aldimines **19** with vinylazaarenes **20** (Scheme 5 C). A combination of CuF(PPh_3_)_2_⋅2MeOH and dppf **L5** afforded, after in situ oxidation, 1,3‐amino alcohols **21** with moderate to high diastereoselectivity (Scheme 5 D).

**Scheme 6:**
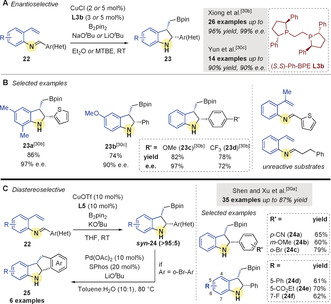
A,B) Xiong's and Yun's enantioselective Cu‐catalyzed borylative couplings of aldimines and tethered aryl alkenes. C) Shan and Xu's diastereoselective variant.

Following these reports, three groups simultaneously reported a related intramolecular coupling (Scheme 6);^[30]^ *N*‐(2‐Vinylphenyl) aldimines **22** were reacted with B_2_pin_2_ in the presence of a copper catalyst to give 2,3‐disubstituted indolines **23**. Both Xiong and co‐workers^[30b]^ and Yun and co‐workers^[30c]^ reported an enantioselective coupling using (*S*,*S*)‐Ph‐BPE **L3 b** (Scheme 6 A). A range of (hetero)aryl substituents on the imines and aryl‐substitutents on the alkene was well tolerated (Scheme 6 B). Shen, Xu and co‐workers^[30a]^ reported a diastereoselective variant using dppf **L5** (Scheme 6 C). Interestingly, the products arising from the reaction of 2‐bromo‐aryl imines were further transformed into the corresponding tetrahydroindenoindoles **25** through a palladium‐catalyzed Suzuki–Miyaura coupling.

### 3.3 Dienes

**Scheme 7:**
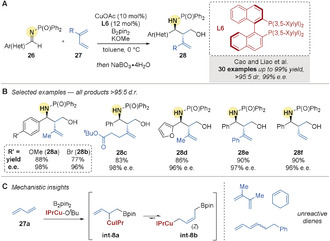
Cao and Liao's enantioselective Cu‐catalyzed borylative couplings of aldimines and dienes.

In 2016, Cao, Liao, and co‐workers^[31]^ reported the copper‐catalyzed diastereo‐ and enantioselective borylative coupling of imines **26** and dienes **27**. Following in situ oxidation, 1,3‐amino alcohols **28** were obtained (Scheme 7 A). Very high diastereoselectivities and excellent enantioselectivities were obtained with the bulky phosphine ligand (*R*)‐DM‐BINAP **L6** (Scheme 7). This three‐component coupling reaction was effective with dienes ranging from the highly abundant 1,3‐butadiene (to give **28 f**) and isoprene (to give **28 a**, **28 b**, **28 d**), to more functionalized 2‐substituted dienes (to give **28 c**). Unfortunately, the reactions with internal, cyclic, and 2,3‐substituted dienes were unsuccessful. The authors were able to isolate the (*Z*)‐allyl copper species **int‐8 b**, which they suggested was formed from initial 1,2‐borocupration of 1,3‐butadiene, followed by migration of copper to the least hindered position (Scheme 7 C). This (*Z*) geometry is often thermodynamically favored in allylmetals.[^32^, ^33^]

**Scheme 8:**
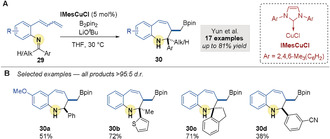
Yun's Cu‐catalyzed intramolecular borylative couplings of aldimines and dienes.

In 2018, Yun and co‐workers^[34]^ reported an intramolecular copper‐catalyzed borylative coupling of 1‐dienylarenes **29** with tethered imines to give 7‐membered benzo[*b*]azepines **30** (Scheme 8 A). Aryl and alkyl aldimines, and aryl, alkyl ketimines, were suitable substrates and gave products with high diastereocontrol (Scheme 8 B).

**Scheme 9:**
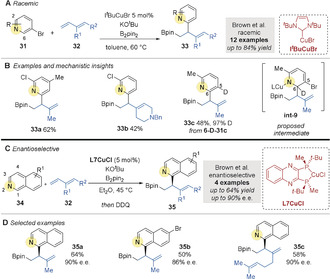
A,B) Brown's Cu‐catalyzed borylative *cine*‐substitution of 3‐bromopyridines. C,D) Brown's enantioselective Cu‐catalyzed borylative couplings of isoquinolines and dienes.

In 2018 Brown and co‐workers^[35]^ serendipitously discovered the borylative *cine*‐substitution of 3‐bromopyridines **31** with dienes **32** (Scheme 9 A). Labelling and crossover experiments suggested a concerted deuterium migration from the 6‐ to the 5‐position in intermediate **int‐9** (Scheme 9 B). Quinolines and isoquinolines did not undergo *cine*‐substitution, but direct addition after borocupration of 2‐alkyl‐1,3‐dienes **32**. For example, direct addition occurred at the 1‐position of isoquinolines and afforded 1‐hydroisoquinolines, which were prone to re‐aromatization upon treatment with DDQ. This transformation was rendered enantioselective using **L7CuCl** (Scheme 9 C,D).

### 3.4 Enynes

**Scheme 10:**
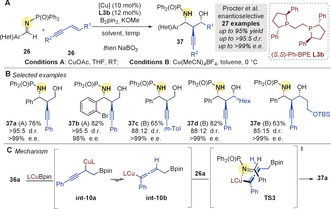
Procter's enantioselective Cu‐catalyzed borylative couplings of aldimines and enynes.

In 2020, Procter and co‐workers^[36]^ used a copper‐catalyzed borylative coupling of enynes **36** and aldimines **26** to prepare homopropargylic‐1,3‐aminoalcohols^[37]^ **37**, containing up to three contiguous stereocenters, with high enantiocontrol (Scheme 10 A). Interestingly, (*S*,*S*)‐Ph‐BPE **L3 b** (*c.f*. Schemes 5 and 6) was again used to impart enantioselectivity. A wide range of aromatic imines and electron‐rich aromatic enynes were well tolerated (Scheme 10 B). Based on a related DFT study involving carbonyl partners,^[15]^ the authors suggested a mechanism involving rearrangement of the propargylic **int‐10 a** to the allenyl copper **int‐10 b** (Scheme 10 C) followed by coupling via 6‐membered **TS3** to give **37**.

### 3.5 Direct Borylation of Imines

The hydroboration of imines affords important α‐aminoboronic acids that are bio‐isosteres of α‐amino acids.^[38]^

**Scheme 11:**
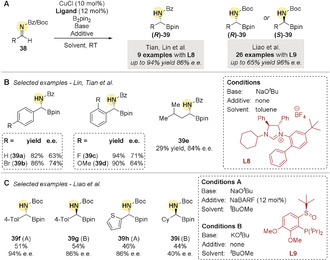
A) Enantioselective Cu‐catalyzed direct hydroboration of aldimines. B) Examples from Lin, Tian et al. C) Examples from Liao, et al.

In 2013, Tian, Lin and co‐workers^[39]^ used *N*‐benzoyl arylaldimines **38** to obtain α‐amido boronic esters **39** in good yields and moderate enantioselectivities (Scheme 11 A,B). In 2015, Liao and co‐workers^[40]^ reported an improved copper‐catalyzed enantioselective hydroboration of *N*‐Boc aldimines **38** using a chiral sulfoxide‐phosphine ligand **L9** (Scheme 11 A,C). Interestingly, the absolute configuration of the resulting α‐amido boronic esters **39** could be controlled by the copper counter ion (Scheme 11 C).

**Scheme 12:**
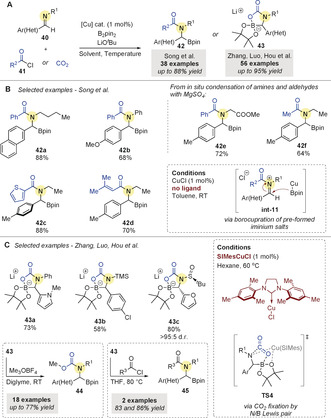
Cu‐catalyzed borylative couplings of aldimines and acyl chlorides, reported by Song and co‐workers, and aldimines and CO_2_, reported by Zhang, Luo, Hou and co‐workers.

The direct borylation of imines has been used in related multicomponent reactions. In 2019, Song and co‐workers^[41]^ reported the copper‐catalyzed boroacylation of aldimines **40** (Scheme 12 A). The two‐step protocol involves formation of the iminium salt **int‐11** then copper‐catalyzed borylation to yield the desired *N*‐acylated α‐amino boronic esters **42**. The process was efficient across a wide range of aryl imines bearing alkyl and aryl *N*‐substituents, and various aromatic and heteroaromatic acyl chlorides (Scheme 12 B). The direct conversion of aldehydes and amines through an in situ condensation/boroacylation sequence (to give **42 e** and **42 f**) was also possible.

Shortly after, Zhang, Luo, Hou and co‐workers^[42]^ reported a highly efficient copper‐catalyzed borylative functionalization of aldimines involving CO_2_ fixation (Scheme 12 A). Following borocupration of the aldimines, intramolecular N/B Lewis pair formation was proposed to efficiently activate CO_2_ (**TS4**; supported by DFT calculations), yielding versatile borocarbamate salts **43**. The methodology was applied to an extensive scope of aryl aldimines using only 1 mol % of a readily available NHC‐ligated copper catalyst (Scheme 12 C). Finally, carbamate‐containing α‐amino boronic esters **44** and **45** were generated upon treatment with a methylating agent or an acyl chloride.

## 4 Copper‐Catalyzed Borylative Couplings with Nitriles

The cyanation of olefins delivers versatile nitrile‐containing products.^[2]^ Electrophilic cyanating agents, such as *N*‐cyano‐*N*‐phenyl‐*p*‐methylbenzenesulfonamide (NCTS **46 a**), have emerged as a safer and more practical alternative to traditional cyanating reagents, such as hydrogen cyanide and cyanide salts.^[43]^ They can be used to intercept nucleophilic organocopper species, and have recently featured as electrophilic partners in copper‐catalyzed borylative MCRs.

### 4.1 Allenes

**Scheme 13:**
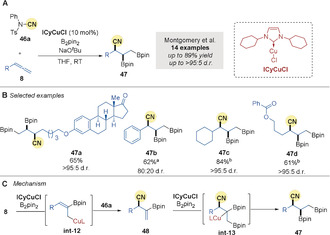
Montgomery's Cu‐catalyzed borylative cyanation of allenes. [a] MeOH was used as additive. [b] standard procedure used then NaOH/H_2_O_2_, overall yield shown for cyanodiboration and oxidation to the diol.

In 2016, Montgomery and co‐workers^[44]^ reported the copper‐catalyzed borylative trifunctionalization of terminal allenes **8** with the cyanating reagent NCTS **46 a** (Scheme 13 A). The process demonstrated high diastereoselectivity, good functional group compatibility, and high yields (Scheme 13 B). As discussed previously (Scheme 2), initial borocupration forms an allyl copper intermediate **int‐12** (Scheme 13 C). Subsequent cyanation with NCTS affords intermediate **48**. A second borocupration, followed by protodemetalation, delivers the trifunctionalized product **47** via **int‐13**.

### 4.2 Vinylarenes

**Scheme 14:**
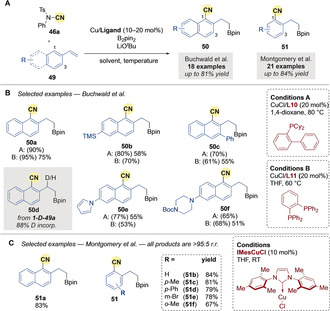
Cu‐catalyzed borylation/*ortho*‐cyanation reported by Buchwald and by Montgomery. Yields in parentheses are crude yields.

In 2014, Yang and Buchwald^[45]^ reported a copper‐catalyzed, regioselective borylative cyanation of 2‐vinylnaphthalene derivatives **49** using NCTS **46 a** (Scheme 14 A). The reaction displayed a clear selectivity for *ortho*‐cyanation over benzylic cyanation (observed in less than 5 %), and an exclusive preference for the most hindered *ortho* position (C1 vs. C3). Substitution at the C6 position of the 2‐vinylnaphthalene derivatives was well tolerated (Scheme 14 B). Interestingly, deuterium labelling and crossover experiments showed an intramolecular transfer of the C1 deuterium of labelled **1‐D‐49 a** to the benzylic position of **50 d**.

Shortly after, Montgomery and co‐workers^[46]^ reported the copper‐catalyzed borylative cyanation of vinylarene derivatives **49** with NCTS **46 a** (Scheme 14 A). The same *ortho*‐selectivity was observed using the copper‐NHC pre‐catalyst IMesCuCl. For 2‐vinylnaphthalene, cyanation at C1 was observed (**51 a**). However, in the case of substituted styrenes, the nitrile group was directed to the least sterically hindered *ortho*‐carbon (Scheme 14 C).

**Scheme 15:**
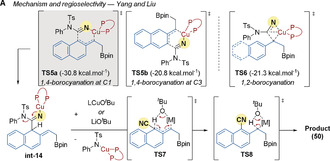
Yang and Liu's computational study of the Cu‐catalyzed regioselective borylation/*ortho*‐cyanation of vinylarenes. M=Cu *or* Li.

The origin of regioselectivity was studied by Yang and Liu^[47]^ using DFT (Scheme 15). They proposed that benzylic cyanation is disfavored as a high energy 4‐membered transition state **TS6** is required, whereas the *ortho*‐cyanation features a more favorable six‐membered TS (**TS5 a** and **TS5 b**). The selectivity for C1 over C3 cyanation in 2‐vinylnaphthalene derivatives comes from a lower disruption of the aromaticity of the naphthalene system in **TS5 a** than in **TS5 b**. Facile 1,2‐elimination of the copper tosylamide from **int‐14** then leads to a dearomatized intermediate. Next, as previously demonstrated by Yang and Buchwald in a deuterium labeling experiment, rearomatization occurs by an intramolecular 1,3‐migration of the C1 hydrogen via a six‐membered transition state **TS7**, followed immediately by benzylic protonation **TS8**.

**Scheme 16:**
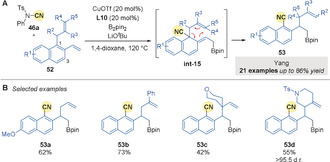
Yang's Cu‐catalyzed 1,3‐allyl group transfer borylation/*ortho*‐cyanation cascade.

Yang^[48]^ recognised the potential of the dearomatized intermediates to undergo rearomatization‐driven sigmatropic rearrangement and disclosed a catalytic borylation/*ortho*‐cyanation/Cope rearrangement sequence (Scheme 16 A). After the initial borocupration and *ortho*‐cyanation (vide supra), pre‐installed allyl chains at the C1 position of 2‐vinylnaphthalene derivatives **52** migrate to the benzylic position in a [3,3]‐sigmatropic Cope rearrangement to re‐establish aromaticity without the need for an alkoxide‐assisted [1,3]‐H shift (see **int‐15**, Scheme 16 A). Neither benzylic cyanation or functionalization at the allyl moiety were observed.

**Scheme 17:**
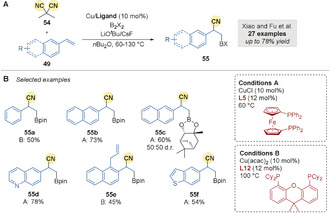
Xiao and Fu's Cu‐catalyzed borylation/benzylic cyanation of vinylarenes.

In 2018, Xiao, Fu and co‐workers^[49]^ reported a complementary copper‐catalyzed borylative benzylic cyanation of vinylarenes **49** (Scheme 17 A). The unprecedented regioselectivity was achieved by using dimethylmalononitrile (DMMN) **54**, instead of NCTS derivatives **46**, as the electrophilic cyanating agent. Benzylic nitriles were obtained in good yields from a wide range of vinylarenes, encompassing 2‐vinylnaphthalene and styrene derivatives (Scheme 17 B). Notably, the presence of an allyl chain at the C1 position of 2‐vinylnaphthalene did not lead to the Cope rearrangement seen by Yang.

### 4.3 Dienes

**Scheme 18:**
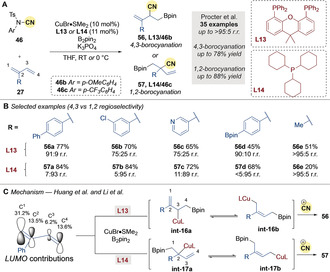
A,B) Procter's Cu‐catalyzed regiodivergent borocyanation of dienes. C) Huang's mechanistic DFT studies.

In 2018, Procter and co‐workers^[50]^ reported the ligand‐controlled, regiodivergent borocyanation of 1,3‐dienes **27** (Scheme 18 A). The bidentate phosphine ligand XantPhos **L13** and 4‐methoxy substituted NCTS derivative **46 b** gave excellent regiocontrol and very good yields of the 4,3‐borocyanated product **56** (Scheme 18 B). Whereas, switching to the monodentate phosphine ligand PCy_3_ **L14** and *para*‐CF_3_ NCTS derivative **46 c** enabled 1,2‐borocyanation to give products **57** with good selectivity and excellent yields (Scheme 18 B).

Shortly after, both Huang and co‐workers^[51]^ and Li and co‐workers^[52]^ investigated the origin of the regiodivergency through DFT calculations (Scheme 18 C). They suggested that, due to the steric bulk of ligand **L13**, borocupration occurs across the less hindered C4−C3 double bond to give the allyl copper species **int‐16 a** followed by rearrangement to **int‐16 b**. Subsequent cyanation gives the 4,3‐borocyanated product **56**. Conversely, borocupration occurs across C1−C2 with the less bulky ligand PCy_3_ **L14** due to the greater contribution of C1 to the LUMO, ultimately leading to the 1,2‐borocyanated isomer **57**.

**Scheme 19:**
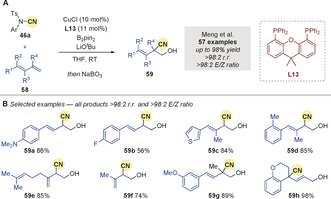
Meng's Cu‐catalyzed regioselective borocyanation of dienes.

Soon after, Meng and co‐workers^[53]^ disclosed the copper‐catalyzed cyanation of substituted 1,3‐diene derivatives **58** (Scheme 19 A). Excellent regioselectivity and *E*‐stereoselectivity was achieved using CuCl and ligand **L13** across a wide range of substrates (Scheme 19 B). 4,3‐Borocyanation dominated with 1‐substituted, 1,2‐disubstituted, and 1,3‐disubstituted dienes yielding products **59** exclusively (see **59 a**–**g**). On the other hand, 1,1‐disubstituted dienes afforded products such as **59 h**, arising from 4,1‐borocyanation.

**Scheme 20:**
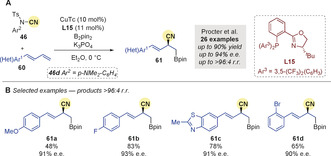
Procter's Cu‐catalyzed regio‐ and enantioselective borocyanation of dienes. CuTc=copper(I) thiophene‐2‐carboxylate.

In the same year, Procter and co‐workers^[54]^ disclosed an enantioselective 1,2‐borocyanation of 1,3‐dienes **60** that delivered enantiomerically enriched allylic nitriles **61** (Scheme 20 A). Substrates bearing heterocycles and substituted arenes were compatible, and gave the anticipated products with excellent regio‐ and enantiocontrol (Scheme 20 B).

### 4.4 Addition Across Nitriles

**Scheme 21:**
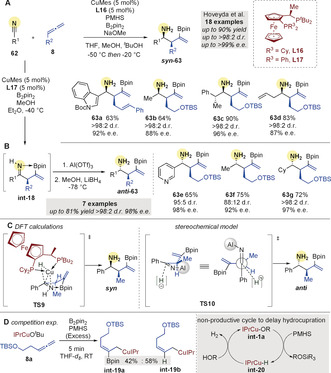
Hoveyda's Cu‐catalyzed borylative couplings of nitriles and allenes. PMHS=polymethylhydrosiloxane.

In 2019, Hoveyda and co‐workers^[55]^ presented a borylative coupling of allenes and nitriles to access primary alkylamines (Scheme 21 A). The process consists of two copper‐catalyzed cycles: first, borocupration of **8** gives an allylcopper species that adds to nitriles **62**, giving ketimine intermediates (*c.f*. **int‐18**). In a second cycle, these intermediates are reduced to the desired amine **63** by a copper hydride species.^[56]^ Alkylallenes were coupled to a range of aromatic and aliphatic nitriles using **L16** to afford ***syn*** **‐63** in good yields, with very good diastereo‐ and enantiocontrol (Scheme 21 A). Intramolecular N/B coordination was shown to be essential to activate the ketimine **int‐18** towards Cu−H reduction and to achieve excellent stereocontrol (**TS9**, Scheme 21 C). Through a three‐step process, ***anti*** **‐63** products were obtained utilizing ligand **L17** (Scheme 21 B). As spontaneous reduction does not occur with this system, Al(OTf)_3_ was added to promote decoordination of the N/B pair and allow LiBH_4_ reduction (**TS10**, Scheme 21 C).

Competition experiments (Scheme 21 D) showed that formation of the copper‐hydride **int‐20**, hydrocupration of the allene **8** and addition of the resulting organocopper species (e.g. **int‐19 b**) to the nitrile were all faster than the equivalent borylative process. To avoid this undesired process, Hoveyda and co‐workers used an excess of the polymeric silane, polymethylhydrosiloxane (PMHS), and a finely tuned mixture of alcohols to engage the copper hydride catalyst in an unproductive cycle (Scheme 21 D).

**Scheme 22:**
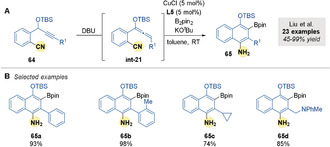
Liu's Cu‐catalyzed borylative cyclization of *o*‐cyanophenyl propargyl ethers.

Prior to this work, Liu and co‐workers^[57]^ had reported a related intramolecular borylative cyclization of *o*‐(cyano)phenyl propargyl ethers **64** (Scheme 22 A). Allenes were first generated in situ by DBU isomerization (**int‐21**). Borocupration by a copper/dppf complex followed by addition to nitriles formed ketimine intermediates that spontaneously isomerized to gain aromaticity and form naphthylamines **65** (Scheme 22 B).

## 5 Copper‐Catalyzed Borylative Couplings with Isocyanates

**Scheme 23:**
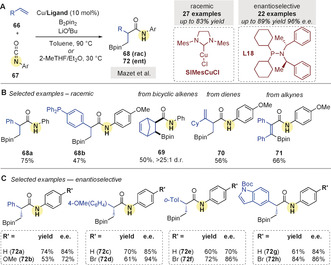
Mazet's Cu‐catalyzed borylative couplings of isocyanates and olefins.

In 2020, Mazet and co‐workers^[58]^ reported the synthesis of chiral β‐borylated secondary amides **68**/**72** from the coupling of styrenes **66** with isocyanates **67** (Scheme 23 A). Good yields were obtained using an achiral NHC ligand (Scheme 23 B). Other olefins were also evaluated as coupling partners: strained bicyclic alkenes, 1,3‐dienes, and alkynes afforded **69**, **70**, and **71**, respectively, in moderate to good yields, however, substituted and heteroaromatic styrene derivatives were not effective. In addition, an enantioselective functionalization of styrenes was developed with the chiral phosphine ligand **L18** (Scheme 23 A,C).

## 6 Summary and Outlook

The past decade has seen significant progress made in the development of a suite of copper‐catalyzed processes for the borofunctionalization of olefins using C−N electrophiles. Numerous olefins, ranging from complex polyenes to simple styrene feedstocks, have been validated as coupling partners. Similarly, various C−N inputs have been utilized. Early transformations using achiral catalysts have rapidly evolved into highly enantioselective processes and the approach now allows efficient, catalytic access to important synthetic building blocks.

A number of challenges face this nascent area of research. For example, copper‐catalyzed MCRs are still limited to activated alkenes and extension of this methodology to unactivated, “simple” alkenes remains a goal for the future. Furthermore, a full understanding of the factors affecting regio‐ and stereoselectivity is needed and will aid our ability to predict reaction outcomes and to systematically develop new regio‐ and stereoselective borofunctionalizations.

## Conflict of interest

The authors declare no conflict of interest.

## Biographical Information

*Fabien Talbot received his BSc from the University of Angers (France) with one year spent at the University of Strathclyde (UK). He then undertook an MSc at the University of Strasbourg (France) and did his research project at the University of Manchester (UK) working on nickel catalysis in Prof. David Procter's research group. In 2017 he stayed in the Procter group for a PhD funded by AstraZeneca and the EPSRC. He is also the recipient of an SCI Scholarship. His research aims to develop new copper‐catalyzed processes*.

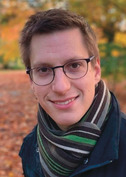

## Biographical Information

*Quentin Dherbassy received his MChem from the University of Strasbourg (France) in 2014 and his PhD in 2017, working in the group of Prof. Françoise Colobert (CNRS, Strasbourg, France). His doctoral studies focused on the control of axial chirality by sulfoxide‐directed C−H activation. After a short post‐doctoral post in the Colobert group, he joined the Procter Group as a PDRA in 2018, and is studying borylative copper‐catalyzed asymmetric multicomponent reactions*.

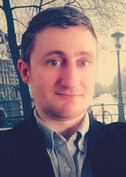

## Biographical Information

*Srimanta Manna received his MSc from IIT Bombay (India) in 2012, and his PhD from the Max Planck Institute of Molecular Physiology, Dortmund (Germany) in 2017. His doctoral studies were carried out in the Antonchick group and focused on hypervalent iodine mediated C−H amination and copper‐catalyzed oxidative cyclopropanation. In 2017, he joined the Procter group and was awarded a Marie Curie Fellowship in 2018. Currently, he is focusing on the development of copper‐catalyzed asymmetric multicomponent coupling reactions*.

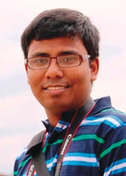

## Biographical Information

*Chunling Shi received her MChem from Jiangsu Normal University (P. R. China) in 2005. In 2009, she obtained her PhD from Southeast University (P. R. China). Her doctoral studies were carried out in the group of Prof. Min Ji on the organocatalytic synthesis of heterocyclic compounds. In 2009, she moved to a Lectureship at Xuzhou University of Technology and was promoted to Associate Prof. in 2013. In 2020, she joined the Procter Group as a visiting scholar*.

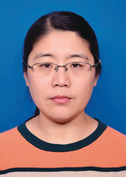

## Biographical Information

*Shibo Zhang received his undergraduate degree from Wuhan Institute of Technology (P. R. China) having carried out research in the area of green chemistry under the supervision of Prof. Zhibing Dong. In 2020, he joined the University of Manchester for his MPhil in the Procter group where he is working on borylative copper‐catalyzed asymmetric multicomponent reactions*.

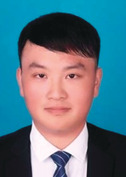

## Biographical Information

*Gareth P. Howell obtained an MChem in 2001 from the University of York including a one‐year internship within Process Chemistry at GlaxoSmithKline. Focussing on asymmetric synthesis and catalysis, he obtained a PhD from the University of Nottingham with Professor James C. Anderson in 2004 before moving to Groningen (NL) to work with Professor Ben L. Feringa as a postdoctoral fellow sponsored by The Leverhulme Trust. He has worked within Process Chemistry at AstraZeneca in the UK since 2006 and is currently an Associate Principal Scientist, leading drug development projects*.

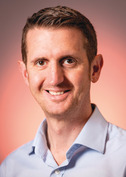

## Biographical Information

*Gregory J. P. Perry obtained his PhD from the University of Manchester (UK) in 2016. His doctoral studies with Prof. Igor Larrosa focused on decarboxylative transformations. In 2017, he moved to Nagoya University (Japan) to work in the group of Prof. Kenichiro Itami, applying C−H activation in chemical biology. Since 2018, Greg has been working as a Lecturer in Organic Chemistry within the group of Prof. David Procter at the University of Manchester (UK)*.

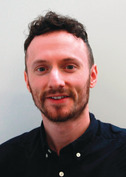

## Biographical Information

*David J. Procter obtained his PhD from the University of Leeds (UK) in 1995 working with Prof. Christopher Rayner on organosulfur and organoselenium chemistry. He then spent two years as a PDRA with Prof. Robert Holton at Florida State University (USA) working on the synthesis of Taxol. In 1997, he took up a Lectureship at the University of Glasgow (UK). In 2004, he moved to the University of Manchester (UK) for a Readership and was promoted to Professor in 2008*.